# Evidence for parasite-mediated selection during short-lasting toxic algal blooms

**DOI:** 10.1098/rspb.2016.1870

**Published:** 2016-10-26

**Authors:** François Blanquart, Myriam Valero, Catharina Alves-de-Souza, Aliou Dia, Frédéric Lepelletier, Estelle Bigeard, Christian Jeanthon, Christophe Destombe, Laure Guillou

**Affiliations:** 1 Department of Infectious Disease Epidemiology, Imperial College London, Norfolk Place, London W2 1PG, UK; 2 Station Biologique de Roscoff, Sorbonne Universités, Université Pierre et Marie Curie (Paris VI), Place Georges Teissier, CS90074, 29688 Roscoff, France; 3 CNRS, UMI 3614, Pontifica Universidad Catolica de Chile, Universidad Austral de Chile, Place Georges Teissier, CS90074, 29688 Roscoff, France; 4 Departamento de Botânica, Museu Nacional, Universidade Federal do Rio de Janeiro, Quinta da Boa Vista, s/n, São Cristovão, Rio de Janeiro, RJ, Brazil; 5 Station Biologique de Roscoff, CNRS, UMR 7144, Place Georges Teissier, CS90074, 29688 Roscoff, France

**Keywords:** coevolution, harmful algal bloom, dinoflagellate, Red Queen, local adaptation, time-shift experiment

## Abstract

Parasites play a role in the control of transient algal blooms, but it is not known whether parasite-mediated selection results in coevolution of the host and the parasites over this short time span. We investigated the presence of coevolution between the toxic dinoflagellate *Alexandrium minutum* and two naturally occurring endoparasites during blooms lasting a month in two river estuaries, using cross-inoculation experiments across time and space. Higher parasite abundance was associated with a large daily reduction in relative *A. minutum* abundances, demonstrating strong parasite-mediated selection. There was genetic variability in infectivity in both parasite species, and in resistance in the host. We found no evidence for coevolution in one estuary; however, in the other estuary, we found high genetic diversity in the two parasite species, fluctuations in infectivity and suggestion that the two parasites are well adapted to their host, as in ‘Red Queen’ dynamics. Thus, coevolution is possible over the short time span of a bloom, but geographically variable, and may feedback on community dynamics.

## 1. Introduction

Host–parasite coevolution, the process of reciprocal adaptive genetic changes in host and parasites [1], shapes levels of diversity [2–5], accelerates the pace of evolution [6], and may drive the evolution of sexual reproduction [7–9] or elevated mutation rate [10–12]. These effects crucially depend on the mode of coevolution: ‘arms race dynamics’ (ARD), where selection is directional and results in escalation of host defence and parasite counter-defence, or ‘fluctuating selection dynamics’ (FSD; also known as Red Queen dynamics), with frequency-dependent selection for rare host and parasite genotypes. ARD results in directional evolution in both partners and increasing infectivity and resistance, and reduces diversity. By contrast, FSD maintains polymorphism, stabilizes infectivity and resistance to intermediate levels, and results in local adaptation of the partner with greater capacity to adapt to the changing selective pressures [13,14].

Evidence of host–parasite coevolution has been found in a number of systems, most often using local adaptation and time-shift experiments. These experiments consist of comparing the fitness of the host and/or parasite population on their local (or contemporaneous) partner population, to their fitness when transferred to partner populations at other locations (or other time points). Interpretation of these experiments has been guided by a number of theoretical predictions under stylized models of coevolution [13–17]. Local adaptation and time-shift experiments have revealed coevolution in diverse biological systems ranging from bacteria–phage to plant–rust and snail–trematode systems [4,17–23]. Evidence of coevolution in eukaryotic species in natural systems is rare, and known examples involve timescales of a few years [19,22]. Both ARD and FSD were detected, and in fact, the same system can shift from one type of dynamics to the other depending on resource abundance or time [18,24–26].

Coevolution supposes that two species coexist in an antagonistic relationship long enough for reciprocal adaptive genetic changes to take place, which seems unlikely in communities with high species turnover, such as planktonic assemblages. Dinoflagellate blooms result from the rapid replication of planktonic vegetative cells lasting a few days to a few weeks. Blooms alternate with the production of sexually induced resting stages that can stay dormant in sediment for months to years [27]. Blooms are initiated by germination of these dormant stages triggered by chemical signals, density of conspecifics, or environmental cues, and are supposedly sustained by clonal replication [28]. In spite of the very short time span of a bloom, there are several reasons to suspect marine dinoflagellates coevolve with their parasites. First, microeukaryotic parasites probably impose a strong selection pressure on dinoflagellates and play a role in the control and collapse of blooms. For example, in the Penzé estuary in France, a rapid weekly succession of dinoflagellate species forming blooms (among which *Alexandrium minutum*) was observed during early summer. Each of the blooms was accompanied by an epidemic of the endoparasitic dinoflagellate *Amoebophrya* spp., whose prevalence sometimes reached up to 40% of *A. minutum* hosts. These specialist parasites caused the eventual demise of each bloom, favouring the emergence of another species forming the following bloom [29]. Second, blooms present a very high genetic diversity and spatial and temporal genetic differentiation [30–32]. The possibility that dinoflagellates and their parasites coevolve over very short time spans is of fundamental importance for our understanding of community dynamics and host–parasite coevolution, but also has significant public health implications. Algal blooms and especially those of the genus *Alexandrium* cause massive faunal mortality and even fatalities to humans, as they cause the paralytic shellfish poisoning syndrome [28].

We investigated the presence and properties of coevolution between the dinoflagellate *A. minutum—*the most abundant dinoflagellate species at the time of study in the two river estuaries we sampled—and its local endoparasites during the short time span of blooms. In 2011, for more than a month we followed two simultaneously occurring *A. minutum* blooms in two French estuaries (Rance and Penzé). Our goal was to answer the following questions. Which parasite species infect *A. minutum*? Do these parasites significantly affect host growth? Is coevolution occurring between the two partners? If yes, what is the mode of coevolution, and is it different in the two estuaries? We answered these questions by monitoring the abundance of the host *A. minutum* and its parasites, isolating and culturing monoclonal strains of both the host and microeukaryotic parasites at several time points in these two estuaries, and conducting a large number of cross-inoculation experiments over time and space to characterize the coevolutionary process.

## 2. Material and methods

### (a) Host and parasite sampling strategy

Samples were collected during two *A. minutum* bloom events occurring early summer 2011 (May–June) in two estuaries distant of each other by approximately 170 km; the Penzé and the Rance estuaries in France (electronic supplementary material, table S1). During this period, seawater samples were collected daily following the sampling strategy detailed in the electronic supplementary material.

### (b) Isolation, identification, and genotyping of host and parasite strains

All strains (hosts and parasites) were grown in F/2 medium (Marine Water Enrichment Solution, Sigma) prepared by autoclaving natural seawater from Penzé (salinity 27 practical salinity units (psu)) collected at least three months prior to use and stored in the dark. This medium was supplemented with 5% (v/v) soil extract [33] and sterilized by filtration using a 0.22 µm pore size filter under sterile conditions. All stock cultures and experiments were conducted at 19°C and on a 12 light (L) : 12 dark (D) cycle at 80 µmol photons m^2^ s^−1^. During the course of the blooms, monoclonal *A. minutum* cultures were established once a week by transferring single cells into fresh medium (one cell in 1 ml individually cultured in 24 well plates, on two to three plates per week).

To isolate parasites at all sampling dates, fresh samples (1 ml) were incubated in a 24 well plate with 1 ml of exponentially growing *A. minutum* culture (13 different strains listed in the electronic supplementary material, table S2). The presence of parasites was screened by microscopy until 15 days of incubation. Two species of Perkinsozoa (*Parvilucifera infectans* and *Parvilucifera rostrata*) were observed. Monoclonal parasite lines were then established using a glass micropipette to transfer a single sporangium into 1 ml of exponentially growing fresh host culture (same strain as that in which the parasite was established). Strains were purified three times using this procedure. During this period, strains were maintained by weekly transfer of 100 µl of infected host culture into 1 ml of exponentially growing host culture. All monoclonal parasite strains were then transferred successfully into a standard host strain, *A. minutum* strain RCC3018, for their maintenance (this host strain, isolated in 1989, could be infected by all parasite strains isolated in the present study).

The host and the parasite species were confirmed by sequencing the intergenic region of ribosomal DNA (ITS1, 5.8S, and ITS2; details in the electronic supplementary material). All strains used in this study (electronic supplementary material, table S2) are deposited at the Roscoff Culture Collection (http://roscoff-culture-collection.org/).

Most *A. minutum* strains used in this study (76/115 strains) were genotyped in a previous study with 12 microsatellite markers [32]. To complete the dataset, 10 additional strains used in this study were genotyped according to Dia *et al.* [32]. Genotypes were grouped into three genetic clusters using the software STRUCTURE v. 2.2 [34] (electronic supplementary material, table S2). STRUCTURE defines cluster based on minimization of Hardy–Weinberg and linkage disequilibrium within clusters.

### (c) Host–parasite population dynamics

Dinoflagellate abundance was quantified using an inverted microscope at a 20× magnification (Nikon Eclipse TS100), after overnight sedimentation of material fixed with Lugol in 10 ml columns [35]. Quantifications were performed in random fields [36] until at least 100 units of the dominant species were enumerated [37].

Dynamics of the parasite populations in Penzé and Rance were assessed by quantitative PCR (qPCR) and the detailed experimental procedures are provided in the electronic supplementary material.

### (d) Cross-infections

**Figure 1.**
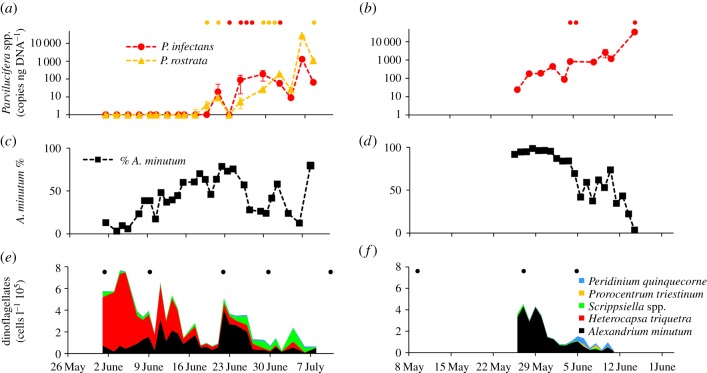
Host–parasite (*A. minutum*-*Parvilucifera* spp.) dynamics. Blooms occurred during early summer 2011 in the Penzé (*a,c,e*) and the Rance (*b,d,f*) estuaries. (*a,b*) Estimation by qPCR of the relative contribution of *P. infectans* and *P. rostrata* to the total bulk of extracted genomic DNA (gDNA) (expressed in copy number per nanogram of DNA ± standard error (s.e.)). (*c,d*) Contribution of *A. minutum* to the total number of dinoflagellates (expressed in % of enumerated cells). (*e,f*) Contribution of the different dinoflagellate species to the total dinoflagellate density (cells l^−1^ 10^5^). The points at the top of panels (*a,b*) and (*e,f*) show the parasite and host sampling times, respectively.

The properties of the coevolutionary process between the toxic microalga *A. minutum* and its microeukaryote parasites were inferred by cross-infections using host and parasite strains isolated at different dates along the blooms (figure 1). In total, we used eight strains of *P. infectans* from Penzé, seven strains of *P. infectans* from Rance, and 22 strains of *P. rostrata* from Penzé. For each species and location, we sampled three to five time points, and one to seven parasite strains per time point. These parasite strains were cross-inoculated with 115 strains of *A. minutum* (eight dates of isolation with 10–18 strains per date, electronic supplementary material, table S2). A total of 3 711 cross-infection assays were conducted (electronic supplementary material, table S3) within 11 months after strain isolation.

The cross-infection assays were conducted as follows. Exponentially growing hosts (cell concentrations > 10^4^ cells ml^−1^) were obtained by diluting 5–10 ml of starter cultures into 30–40 ml of fresh medium once a week and for at least three consecutive weeks prior to the experiment. Freshly produced parasite zoospores (at concentration ≥ 10^5^ zoospores ml^−1^) were obtained by transferring 500 µl of infected culture into 5 ml of exponentially growing culture of *A. minutum* strain RCC3018. After 3–5 days of incubation, the zoospores produced were filtered through a 5 µm cellulose acetate membrane (Minisart, Sartorius, Germany) to remove remains of the initial host. Aliquots (100 µl) of zoospores were then inoculated into 1 ml of exponentially growing host cultures. Results of cross-infections were recorded by microscopic inspection of the host population after 10 days. Hosts were considered as sensitive when sporangia and less than 10 swimming host cells were observed and as resistant otherwise. The test was not repeated when all host cells had been killed after the first incubation period. Alternatively, biological replicates were conducted generally within two weeks following the first assay. Variability between these biological replicates can be caused by uncontrolled environmental or experimental variability, differences between the different generations of the host and parasite strains, and stochasticity in the outcome of the experiment. Of the 614 tests performed in triplicate, 71% gave identical results for all three assays, implying the probability of the observed outcome in a single experiment was 89%. Thus, these potential sources of variation contributed very little variability. For each combination of host–parasite strain, we defined a single result, ‘infection’ or ‘no infection’, as that obtained most often in the three assays.

### (e) Statistical analyses

To quantify the impact of parasites on host population dynamics, we regressed the change in fraction of *A. minutum* per day over the population size of *P. infectans* and *P. rostrata*. Conversely, to quantify the impact of hosts on parasite population dynamics, we regressed the change in the number of parasites per day over the host population size. Significance of the regression coefficients was assessed using *t*-tests.

We performed five separate statistical analyses on the cross-inoculation experiments. For clarity, we numbered these analyses from (i) to (v) and used the numbers in the corresponding ‘Results’ section.

(i) We tested whether infectivity matrices were nested. A nested infectivity matrix means that while the most generalist parasite strain infects a large subset of host strains, more specialist parasites infect successive nested subsets of the host strains. A nested matrix is suggestive of a ‘gene for gene’ model of coevolution [38]. To quantify nestedness, we used ‘temperature’, a measure ranging from *T* = 0 for a perfectly nested matrix to *T* = 100 for a modular (‘chessboard’) matrix. Temperature was calculated with the bipartite package in R [39]. Significance was assessed using the distribution of temperature in 10^3^ matrices with the same average infectivity but where the [host strain] × [parasite strain] combinations that resulted in infection were chosen at random.

(ii) We next investigated the specificity of the host–parasite relationship. More specifically, we tested whether host resistance was specific to each parasite species; whether host resistance was specific to the origin of the parasite strain of *P. infectans*; and whether parasite infectivity was specific to host origin. To this end, using linear regression, we tested for an association between resistance to *P. rostrata* and resistance to *P. infectans* for all host strains from the Penzé (the estuary where the two parasite species coexist); between resistance to Penzé *P. infectans* and resistance to Rance *P. infectans* for all host strains; and tested for an association between infectivity on Penzé hosts and infectivity on Rance hosts, for all *P. infectans* strains, and for all *P. rostrata* strains. A positive relationship indicates universal mechanisms of resistance or infectivity (effective across species and estuaries), while a negative relationship indicates a trade-off in the ability to resist or infect different species or strains from different estuaries. No significant relationship indicates resistance or infectivity are independent across species and estuaries. Significance of the regression coefficient was assessed using a *t*-test.

Local adaptation could be tested only for the parasite *P. infectans*, as *P. rostrata* was present only in the Penzé estuary. To make meaningful comparisons across estuaries, in the following, time will be in days relative to the peak of the bloom (i.e. day 0 is 22 June 2011 in Penzé and 27 May 2011 in Rance). We used the information on allopatric cross-inoculations in two ways.

(iii) First, we investigated whether there were differences in average host resistance and parasite infectivity across locations. To this end, we calculated the average infectivity for the four (host origin) × (parasite origin) combinations, and fitted a linear model explaining average infectivity as a function of host origin and parasite origin. Significance was assessed with a type II analysis of variance.

(iv) Second, we tested for local adaptation, which is a component of the host × parasite interaction that emerges when the host or the parasite are adapted to their local partner. Local adaptation is a direct test of host–parasite coevolution; models predict that the partner with the greater capacity to adapt has positive local adaptation, while the other has negative local adaptation [13,14,40]. We considered the strict criterion for local adaptation [41], whereby the metapopulation was considered locally adapted if the fitness of the local population was greater than that of the foreign population, for both estuaries (this test can be done both for the host and for the parasite).

(v) To get a comprehensive picture of the temporal dynamics of infectivity and resistance, we used linear modelling of the temporal trends in infectivity and resistance [17]. Specifically, we used a generalized linear model with binomial response to model infectivity as a function of the date of the host, the date of the parasite, the host's genetic cluster, and a time-shift effect. The effect of the host date represents temporal fluctuations in host resistance; the effect of the parasite date represents temporal fluctuations in parasite infectivity. The time-shift effect quantifies the capacity of the parasite to infect ‘far past’, ‘near past’, ‘present’, ‘near future’ hosts, corresponding to time shifts of less than −30 days, −30 to −10 days, −10 to +10 days, +10 to +30 days between the parasite and the host. The time-shift effect emerges from host × parasite interactions for fitness. Fitting host date, parasite date, and the time-shift, together, allows distinguishing between FSD and ARD [17]. Typically, under FSD, parasite infectivity and host resistance do not necessarily change with time, which is why it was originally proposed to use time-shift experiments to reveal FSD coevolution [19]. Indeed FSD results in a time-shift pattern whereby parasite infectivity is higher in the contemporary hosts than in hosts of the past and the future, if parasites adapt faster than the host. By contrast, ARD results in directionally increasing host resistance and parasite infectivity, but does not necessarily result in a time-shift pattern [17]. Lastly, to account for within-population variability in parasite infectivity and host resistance, we included a random effect for the parasite strain and the host strain. The variance of the parasite strain random effect represents genetic variability in the parasite, and similarly for the host. These two variances are assumed to be constant over time.

We fitted this model separately for each location and each type of parasite species (*P. infectans* in Rance, and *P. infectans* and *P. rostrata* in Penzé). We fitted dates of both host and parasite as categorical variables and continuous linear variables, but retained the analysis with dates as categorical variables because it fitted better in most cases in terms of Akaike Information Criterion (AIC). Significance of fixed effects was tested using a type II analysis of deviance (Wald *χ*^2^-test). Significance of random effects was tested using a likelihood ratio test. All effects reported here refer to the full linear model and are therefore adjusted for the other covariates of the model. There was a high rate of false positive regarding the detection of temporal trends in infectivity in parasites, certainly because of the very small number of parasite strains sampled. We thus corrected the *p*-values using randomized pseudo-datasets (electronic supplementary material). In addition, we tested for effects of date host and date parasite using a simpler one-way analysis of variance for the resistance of host by date (when averaged over all parasite strains), and for the infectivity of the parasite by date (when averaged over all host strains).

## 3. Results

### (a) *Alexandrium minutum* and parasite population dynamics

Blooms of the toxic dinoflagellate *A. minutum* started in early summer and lasted one month in both estuaries (figure 1). Maximal *A. minutum* densities reached 4.0 × 10^5^ cells l^−1^ on 22 June 2011 and 4.3 × 10^5^ cells l^−1^ on 27 May 2011 in Penzé and Rance, respectively (figure 1). In Penzé, *A. minutum* was the dominant species from 15 to 27 June. *Heterocapsa triquetra* was dominant during the first two weeks of June, followed by *Scrippsiella* spp., *H. triquetra*, and *Peridinium quinquecorne*. By contrast, *A. minutum* was, most of the time (26 May to 11 June), the dominant species in Rance.

Two species of microeukaryotic parasites, *P. rostrata* and *P. infectans*, infected *A. minutum* [42]. Both *Parvilucifera* species were detected by qPCR in Penzé, while only *P. infectans* was detected in Rance. In line with this result, *P. infectans* was the only parasite isolated from Rance. The two parasites were not detected by qPCR at the beginning of *A. minutum* blooms, but, as *A. minutum* reached high proportions, the abundance of the parasites increased exponentially until the end of the survey in both estuaries (figure 1). In Penzé, abundance of both species exhibited similar fluctuations, suggesting that common factors such as host abundance, host resistance, or other abiotic factors, drive the abundance of the two species.

**Figure 2.**
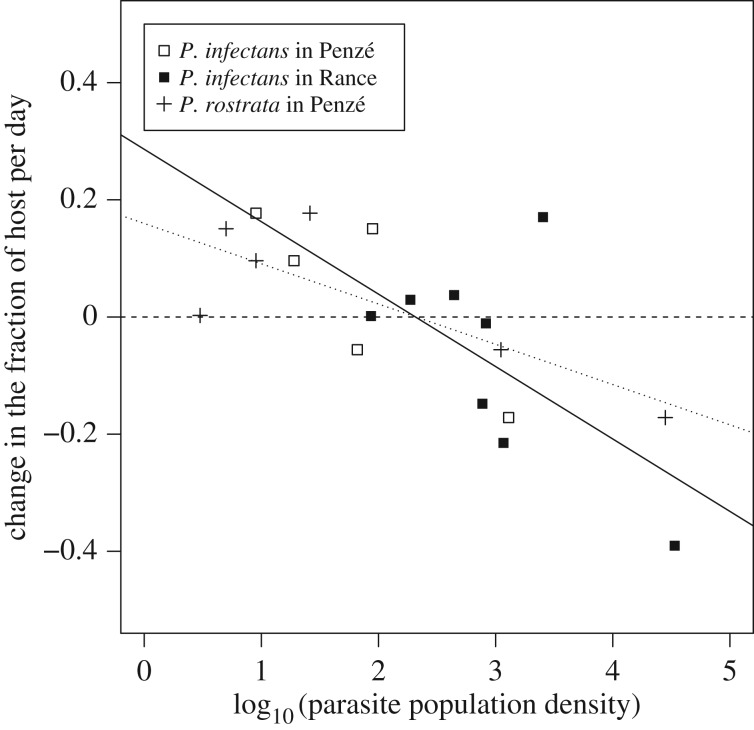
Change, per day, in the contribution of the host *A. minutum* to the total number of dinoflagellates, as a function of parasite population density, for *P. infectans* in Penzé, *P. infectans* in Rance, and *P. rostrata* (present only in Penzé). The solid line is the linear regression for *P. infectans* (Penzé and Rance together, as the slope of the regression was not significantly different between the two estuaries) and the dotted line is the linear regression for *P. rostrata*.

The two parasite species were important for host population control: there was a negative relationship between abundance of parasite species and changes in the relative contribution of *A. minutum* to the community of dinoflagellate species (regression coefficient −0.12 ± 0.038, *p* *=* 0.0074 for *P. infectans*, −0.068 ± 0.024, *p* *=* 0.051 for *P. rostrata*; figure 2). By contrast, the change in the number of parasites was not significantly correlated with host population size, in particular because the parasite populations kept increasing towards the end of the bloom (−0.206 ± 0.36 for *P. infectans* in Penzé, *p* = 0.580; 0.17 ± 0.47 for *P. infectans* in Rance, *p* = 0.74; −0.24 ± 0.36 for *P. rostrata* in Penzé, *p* = 0.51). However, the fact that abundance of these parasites in both estuaries only increased after *A. minutum* grew indicates their preference for this host during the sampling period (although the two parasites may also infect other dinoflagellate species in cultures [42]). Thus, there was reciprocal selection, a necessary condition for coevolution to occur in these populations.

### (b) Genetics of infection

In the following, numbers (i)–(v) refer to the results of the five separate statistical analyses of the cross-inoculation experiments introduced in the ‘Material and methods’ section.

**Figure 3.**
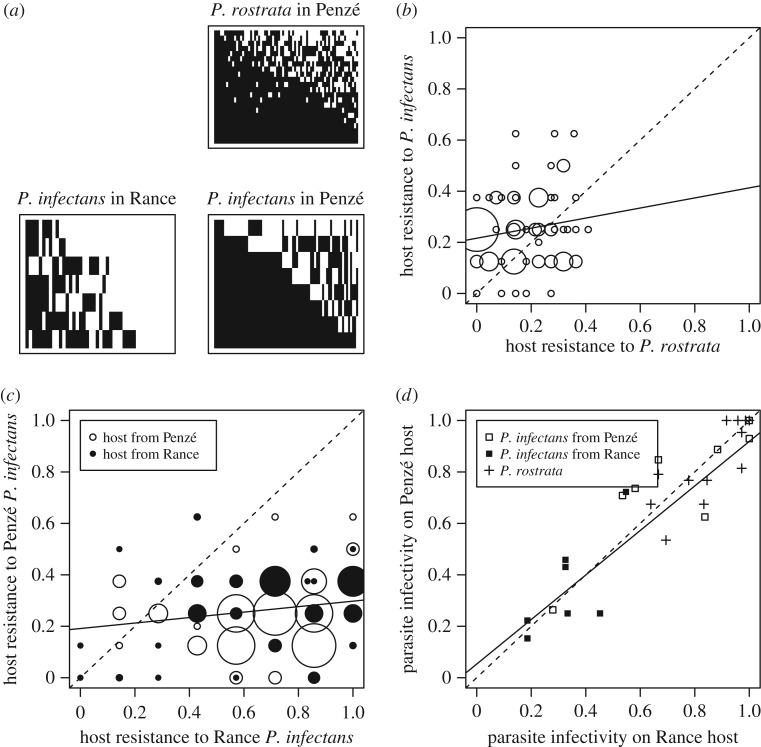
Genetics of interaction between the host and its parasite. (*a*) Infectivity matrices for the two parasites and the two estuaries (*P. rostrata* is absent from the Rance estuary). Parasite strains are in rows, and host strains in columns. Combinations resulting in infection are in black. (*b*) Resistance to *P. infectans* versus resistance to *P. rostrata* for all host strains from the Penzé estuary. (*c*) Resistance to *P. infectans* from Penzé versus resistance to *P. infectans* from Rance for all host strains. (*d*) Parasite infectivity on Penzé hosts versus Rance hosts for all *P. infectans* strains (squares), and for all *P. rostrata* strains (crosses). In (*b–d*), the plain line is the linear regression and the dashed line is the *y* = *x* line. The size of the symbols is proportional to the number of strains with this combination of values.

(i) The capacity of parasite and host strains to infect and resist were both variable (figure 3) and organized in nested patterns suggestive of a ‘gene for gene’ (GFG) model of coevolution. Some parasite strains were able to infect all host strains, and conversely one host strain was infected by all parasites. Infection matrices were significantly nested for both species and in both estuaries (figure 3*a*; electronic supplementary material, table S4).

(ii) Host resistance appeared specific to the parasite species but less so to parasite origin. By contrast, parasite infectivity was universal with respect to the host origin. There was no significant relationship between resistance to *P. infectans* and *P. rostrata* (*R*^2^ = 0.025, *p* *=* 0.18; figure 3*b*). This suggests that resistance to the two parasites was mediated by independent mechanisms. Across spatial locations, the resistance of the host strains to *P. infectans* strains from Penzé versus from Rance was weakly correlated (*R*^2^ = 0.034, *p* = 0.048; figure 3*c*). By contrast, the infectivity of both *P. infectans* and *P. rostrata* parasites to hosts coming from Penzé versus from Rance was strongly correlated (*R*^2^ = 0.80, *p* = 5.9 × 10^−6^, for *P. infectans*; *R*^2^ = 0.69, *p* *=* 2.5 × 10^−4^ for *P. rostrata*, figure 3*d*), suggesting that similar mechanisms mediated infectivity to the hosts of the different estuaries, for both parasites.

(iii) *Parvilucifera infectans* strains from Penzé were more infectious than those from Rance (as can also be seen on figure 3*d*). The average infectivity of strains from Penzé was 0.39 ± 0.038 higher than the infectivity of strains from Rance (*p* = 0.005). However, infectivity did not vary by host origin (*p* = 0.085) or parasite species (*p* = 0.62).

(iv) We detected no local adaptation in *P. infectans*, as the dominant signal was the lower infectivity of Rance parasites (electronic supplementary material, figure S1 and table S5).

### (c) Coevolutionary dynamics in Rance and in Penzé

**Figure 4.**
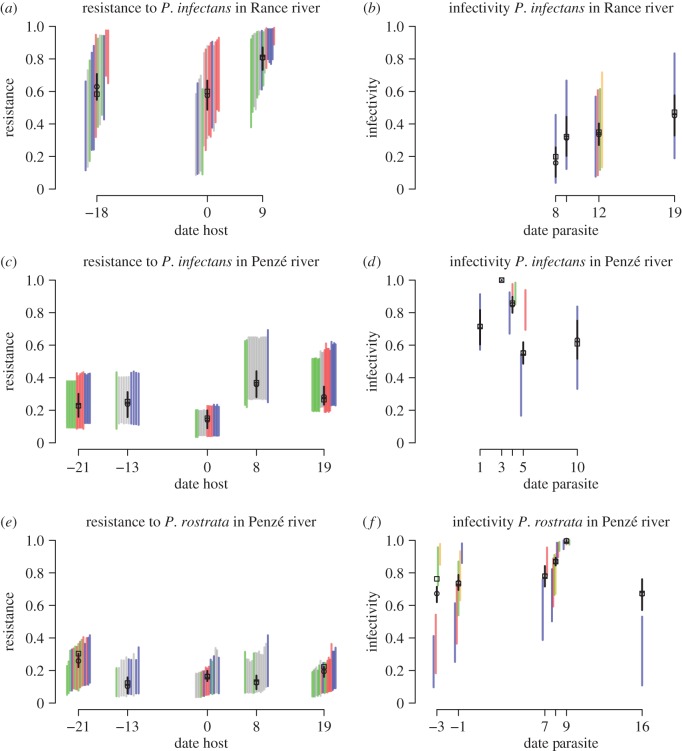
Evolution of the host and parasite, *P. infectans* in the Rance estuary (*a,b*), *P. infectans* in the Penzé estuary (*c,d*), and *P. rostrata* in Penzé estuary (*e,f*). We show resistance as a function of the host date (*a,c,e*), and infectivity as a function of the parasite date (*b,d,f*). In each graph, the average resistance or infectivity in the data for each date (open square) is shown together with the predictions from the linear model (open circle) and 2.5–97.5% bootstrap confidence intervals. The generalized linear model is in very good agreement with the data. Coloured segments illustrate within-population variability in resistance or infectivity. They represent the 95% confidence interval for the best linear unbiased predictor of resistance or infectivity of each individual strain, against one of the parasite strains (for the host) or one of the host strains (for the parasite). Confidence intervals were obtained using a parametric bootstrap where the residual errors were resampled. Resistance of individual host strains is shown against a parasite strain sampled at day 9 for the top row, day 1 for the middle row, and day 8 for the bottom row. Host strains are arranged by increasing resistance and coloured by genetic clusters (grey: unknown cluster). Parasite strains are arranged by increasing mean infectivity and coloured by strain. Infectivity of individual parasite strains is shown against a host strain sampled at day 0 (*a,b*) or day −21 (*c*–*f*).

(v) We used the generalized linear model to investigate the properties of the coevolutionary process in the two estuaries. In the Rance estuary, where only *P. infectans* is present, the host presented a trend of increased resistance (*p* = 0.082; figure 4*a,b*; all effects and *p*-values are reported in the electronic supplementary material, table S6). Resistance did not change from day −20 to day 0, and then increased from 0.6 to 0.8 from day 0 to day 10, corresponding to the period where *P. infectans* became more prevalent in the Rance estuary (figure 4*a*). Resistance did not vary with host genetic cluster (*p* = 0.33), parasite date (*p* = 0.41), or time-shift (*p* = 0.62). Population variability, as quantified by the variance of the random effects in the linear model, was lower in the parasite population (variance = 0.28 on logit scale) than in the host population (variance = 2.01 on logit scale). These observations suggest that in Rance, the host adapted to its parasite population on a time scale of approximately 10 days (about three host generations), but coevolution did not take place, perhaps because the parasite exhibited little genetic variation upon which selection could act.

Coevolutionary dynamics in Penzé were very similar for the two parasite species and suggestive of FSD (figure 4*c–f*). The host exhibited temporal variation in resistance (*p* = 0.024 for resistance to *P. infectans*, *p* = 0.12 for resistance to *P. rostrata*). Although temporal changes in resistance to *P. rostrata* were not significant at the 0.05 level in the generalized linear model, average resistance across all *P. rostrata* strains varied significantly by date according to the one-way ANOVA (*p* = 0.0058, electronic supplementary material, table S6). For the parasites, sampling of a small number of strains meant we had very little power to detect temporal variation and indeed infectivity was never found to significantly fluctuate (electronic supplementary material, table S6). Both parasites were characterized by high diversity as represented by the variance of the ‘parasite strain’ random effect (variance = 2.29 for *P. rostrata*, 0.759 for *P. infectans*, on logit scale), and coexistence of distinct types of strains characterized by low or high infectivity (figure 4*d,f*). Moreover, strains able to infect all host strains emerged in the two parasites (figure 4*d* day 3, figure 4*f* day 9). In both parasites, infectivity subsequently declined, suggesting very high and generalist infectivity carried a fitness cost and could be counter-selected. The host's variability was lower (variance = 0 on the logit scale for resistance to *P. infectans*, 0.63 for resistance to *P. rostrata*) and resistance was not different across genetic clusters (*p* = 0.97 for resistance to *P. infectans*, *p* = 0.73 for resistance to *P. rostrata*).

**Figure 5.**
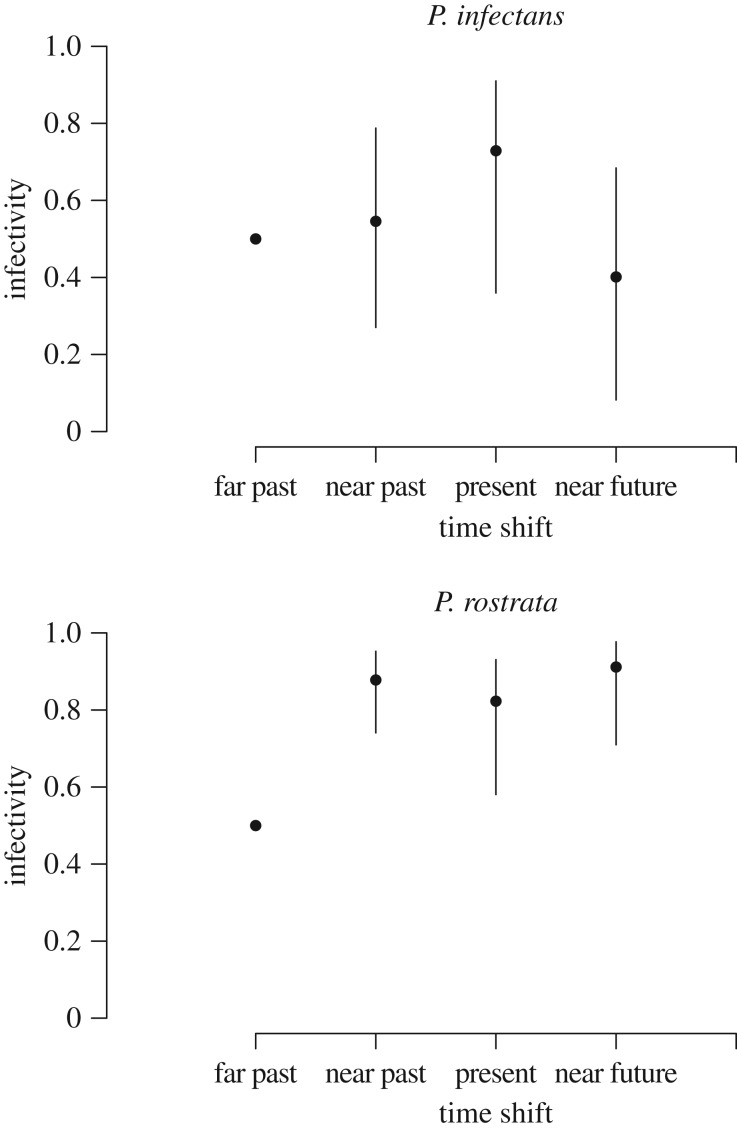
Time-shift effect on the infectivity of parasite *P. rostrata* and *P. infectans* in the Penzé estuary. Time shifts were categorized in far past (less than −30 days), near past (−10 to −30 days), present (−10 to 10 days), and near future (10–30 days). Points are the effects estimated by the linear model, with bootstrap confidence intervals, back-transformed to the probability scale for a host × parasite strain combination of intermediate infectivity 0.5. Time-shift ‘far past’ is taken as reference in the linear model.

The lack of a directional temporal trend in infectivity and resistance, within-population variability, and evidence for temporal fluctuations in infectivity and resistance, all point towards FSD. The time-shift effect presented a non-significant trend of positive adaptation to the contemporaneous host, also suggestive of FSD (figure 5; significance level of the time-shift effect, *p* = 0.06 for *P. infectans*, *p* = 0.096 for *P. rostrata*).

In sum, although resistance to both parasites was not correlated and may therefore operate through different mechanisms, the two species exhibited similar coevolutionary dynamics characterized by high genetic diversity in infectivity, apparition of generalist strains able to infect all host strains, and replaced later by less infectious strains.

## 4. Discussion

The naturally occurring parasites *P. infectans* and *P. rostrata* exerted strong selection on the dinoflagellate *A. minutum* during the few weeks of a bloom, and we found evidence for ongoing coevolution in one of two estuaries. Nested infection matrices and universal mechanisms of infectivity and resistance (figure 3*c,d*) were in line with the GFG model of coevolution.

In the Penzé estuary, we observed large and rapid shifts in resistance, especially for resistance to *P. infectans* between days 0 and 8 (figure 4*c,e*). The host in Penzé had no genetic variability for resistance to *P. infectans* (electronic supplementary material, table S6), implying that evolution must have occurred by selective sweeps, or, more likely, by activation and clonal growth of other genotypes in dormancy, over two to three generations (generation time is about 3 days for the host and the parasite). This seems extremely rapid, but selective pressures are very strong: the host population is wiped out in 10 days by the parasite in the cross-inoculation assays (Material and methods), and the fraction of *A. minutum* among all dinoflagellate species can be reduced by as much as 30% in a day (figure 2). Interestingly, in the Penzé estuary both parasites had large genetic diversity, and evolved highly infectious strains replaced later on by less infectious strains (figure 4*d,f*). These observations are in line with a GFG model of coevolution where high infectivity is costly. This model leads to fluctuating resistance and infectivity and maintenance of genetic variability [43].

The infectivity of *P. infectans* was much lower in the Rance estuary than in Penzé, which may explain the contrasted evolutionary dynamics observed in two estuaries. Low infectivity could be the endpoint of the coevolutionary dynamics, as GFG coevolution can lead to a state where variability is maintained in the host, but the parasite evolves to low infectivity and low variability [43]. This hypothesis supposes a more ancient history of coevolution in Rance leading to this stable state, while coevolutionary fluctuations still occur in Penzé. We did not have any information on the history of parasite presence in Rance, but in Penzé *Parvilucifera* spp. have been intermittently present since at least 1997 [29,44]. Alternatively, the larger infectivity in Penzé could be due to the fact that two parasite species were present, thus intensifying competition, or to the higher nutrient level of this river, one of the most eutrophic of France, which may affect coevolutionary dynamics [24,25].

The biology of the dinoflagellate and parasite species, and the unpredictable and transient nature of blooms, implied we could not freeze the host and parasite samples to conduct the time-shift experiment, and we had limited sampling of the parasite population. We isolated single clones and cultured them alone (for the host) or on a standard host clone (for the parasites), and did all cross-inoculation experiments between 3 and 11 months after isolation. During this period, representing 30–100 generations, new mutations conferring adaptation to laboratory culture conditions or to the standard host clone may have emerged. But this is unlikely to have biased our results, as adaptation to culture conditions would have proceeded similarly across the different strains.

Analysis of this dataset was guided by predictions derived under simple models of coevolution. However, we did not use all the information contained in the cross-inoculation experiments, and in particular the allopatric transplants. Better statistical methods should be developed to more fully interpret this type of experiment. Moreover, some features of this dataset were not considered in most coevolution models. Here, the host was simultaneously infected with two parasite species. Resistance to one species was independent of resistance to the other (figure 3*b*), which may imply that coevolution with the two species could occur simultaneously and independently. The parasites possibly infected other hosts emerging towards the end of the *A. minutum* bloom, particularly in the Penzé estuary (figure 1*e*). In that case, reciprocal selection between parasites and *A. minutum* would be weaker, *A. minutum* would evolve lower resistance to the parasites if resistance is costly, while the evolution of parasite infectivity to the new preferential host would indirectly impact infectivity in *A. minutum.* Host switching may have contributed to the decline in parasite infectivity in *A. minutum* observed in the Penzé estuary towards the end of the bloom (figure 4*d,f*). Such phenomena are fascinating but we had limited power to investigate them here.

To conclude, parasites were important to control blooms of the toxic dinoflagellate *A. minutum*. We revealed high variability in resistance and infectivity and patterns of rapid temporal change, some of them consistent with ongoing host–parasite coevolution. Thus, coevolution is possible over the short time span of a microalgal bloom. However, we did not investigate the reciprocal effect of coevolution on population dynamics. This invites follow-up studies to determine how coevolution affects bloom dynamics, and may shape the rapid succession of blooms of different species observed in these dinoflagellate communities.

## Supplementary Material

Supplementary text and tables

## Data accessibility

Blanquart F, Valero M, Alves-de-Souza C, Dia A, Lepelletier F, Bigeard E, Jeanthon C, Destombe C, Guillou L (2016) Data from: Evidence for parasite-mediated selection during short-lasting toxic algal blooms. Dryad Digital Repository. http://dx.doi.org/10.5061/dryad.731fm [45].
