# Supplementary material for: Evidence for parasite-mediated selection during short-lasting toxic algal blooms
Source: Proc Biol Sci. 2016 Oct 26;283(1841):20161870. doi: 10.1098/rspb.2016.1870 (PMC5095388; doi:10.1098/rspb.2016.1870)

Evidence for parasite-mediated selection during short-lasting toxic algal blooms

François Blanquart, Myriam Valero, Catharina Alves-de-Souza, Aliou Dia, Frédéric Lepelletier, Estelle Bigeard, Christian Jeanthon, Christophe Destombe, Laure Guillou. September 2016.

Electronic Supplementary Material

Sampling strategy

Tidal range inside the Penzé estuary fluctuates from 1.8 to 9.2 m. Because this estuary is relatively shallow (depth lower than 4 m mostly), the sea penetrates deeply (about 6 km) into the flooded valley. The Penzé river flow is rather modest, with a mean annual flow of 4 $m^3.s^{-1}$. The Penzé estuary is well-mixed, meaning that similar salinity is detected from the surface to the bottom and a continuous salinity gradient, ranging from freshwater to marine waters, is detected from upstream to downstream. Plankton samples were collected at 1 m depth using a Niskin bottle, two hours before the high tide at salinities 27 and 29 (psu), reported to display the maximal abundances of *A. minutum* (Maguer *et al.* 2004). In 2011, maximal *A. minutum* densities were detected at salinity 29 (in 80% of samplings).

The Rance estuary is larger (16 km long and 2.5 km wide) but its flow is significantly altered downstream by a tidal power station and upstream by the Châtelier lock which limits the southward progression of seawater. Ten fixed stations were defined along the Rance estuary (Sup. Table 1). Six on ten stations have been sampled every day. Their locations were daily readjusted according to the maximal *A. minutum* density measured the day before in order to maximize the chance to sample the maximal density of the microalga (in 80% of cases, maximal *A. minutum* densities have been detected at stations 3 and 4). Samples were collected two hours before gate aperture of the tidal power station. Plankton samples were collected using a Niskin bottle at the deep chlorophyll maximum (from 0.5 to 4 m depth), determined on board using a portable CTD (CastAway, ISY).

For microscopic analyses, 50 mL of the sample were fixed on board by 2% Lugol's solution. For molecular studies, seawater samples (1-2 L) were fractionated in the laboratory by sequential filtration through 47 mm diameter polycarbonate filters with 10 and 3 μm (Whatman) pore sizes and a

0.2-µm-pore-size Sterivex unit (Millipore). Filters were submerged in lysis buffer (0.75 M sucrose, 50 mM Tris-HCl, pH 8) and immediately frozen in liquid nitrogen and stored at -80°C. The rest of the fresh sample (3-5 L) was immediately processed for strain isolation.

Details of the DNA extraction and PCR reactions

1. Identification of host and parasite species

DNA extraction was performed on strains collected during their exponential growth stage. The dinoflagellate host was directly centrifuged, while the parasite zoospores were filtered through a 5-µm cellulose acetate filter (Minisart, Sartorius, Germany) to remove all remains of the initial host, then centrifuged.

DNA of the dinoflagellate host and the parasite strains was extracted using either the CTAB method (Lebret *et al.* 2012) or the Nucleospin 96 Plant kit (Macherey-Nagel, Düren, Germany), according to the manufacturer's instructions, using 5–10 mg of dried tissue resuspended in 100 µL elution buffer. PCR reactions (14 µL in total) included 20 ng of DNA, 1X GoTaq Flexi Green buffer (Promega, Madison, WI, USA), 150 µM of each dNTP (Thermo Fisher Scientific Inc., Waltham, MA, USA), 20 pmol of the forward primer 329-F (5'-GTG AAC CTG CRG AAG GAT CA-3', complementary reverse of the eukaryote reverse primer 329-R (Moon-van der Staay *et al.* 2001), 20 pmol of the reverse primer DIR-R (5'- TAT GCT TAA AAT TCA GCA GGT-3' (Scholin & Anderson 1994) and 1.25 U GoTaq® Polymerase (Promega). Amplification was carried out in a DNA Engine Peltier Thermal Cycler (Bio-Rad, Hercules, CA, USA) with the following cycling conditions: an initial denaturation at 95 °C for 5 min, followed by 35 PCR cycles (denaturation at 95 °C for 45 s, annealing at 53 °C for 45 s and extension at 72 °C for 45 s), followed by a final extension at 72 °C for 10 min. The PCR products were purified using the BigDye® Terminator v3.1 Cycle Sequencing kit (Applied Biosystems, Foster City, CA, USA) for subsequent bidirectional sequencing using a capillary sequencer type ABI 3130XL (Applied Biosystems).

The endoparasite *Amoebophrya* spp., which commonly infects dinoflagellates, was not observed infecting *A. minutum* during this study. We screened for its presence using fresh samples by

epifluorescence microscopy (BX51, Olympus) equipped with the U-MWB2 cube (450- to 480-nm excitation, 500-nm emission, (Coats & Bockstahler 1994). However *Amoebophrya* spp. infected the other dinoflagellates *Scrippsiella* spp. and *H. triquetra*.

2. Quantification of parasite species by qPCR

For environmental DNA extraction, filters were thawed at 37 °C and incubated for 20 min in presence of 70 µL of lysozyme (50 mg mL^{-1}). Then 50 µL of proteinase K (10 mg mL^{-1}) and 350 µL of sodium dodecyl sulfate (SDS) 10% were added. The mixture was incubated 20 min at 37 °C and the reaction was stopped by 10 min at 55 °C. Total gDNA was extracted using a vol/vol mixture of phenol:chloroform:isoamyl alcohol (25:24:1) and the tubes were gently agitated and incubated for 15 min at 55 °C. A 15-min centrifugation step at 4,500 g at 12°C allowed the recuperation of the aqueous phase. This step was repeated twice. Genomic material was purified and concentrated using Amicon Ultra 4 (Millipore Corporation, USA) following the manufacturer's recommendations. DNA concentration was estimated by using the Quant-it Picogreen dsDNA Assay kit (Invitrogen, Eugene, OR). Environmental DNA extracts were then stored at -80 °C until used. All DNA templates were readily amplified by PCR using general eukaryotic primers (forward primer= GTGCCAGCASCYGCGGTAA, reverse primer= TTTAAGTTTCAGHCTTGCG), so no noticeable inhibition was detected. *Parvilucifera* species abundance was monitored using two sets of specific primers (for *P. rostrata* Par1qF= CACTCTATGTTCCACCGTCGT; Par2qR= GTTCGCTGCGTCCTTCAT and for *P. infectans* Par7qF= AATACGATGAAGGACGTGGC; Par8qR= CCGACCCCTATGAAGAGACA). The presence of these species in DNA extracts was first evaluated by PCR using the following thermal cycling conditions: 95°C for 15 min, 35 cycles of denaturation at 95°C for 1 min, annealing temperature at 57°C for 75 s, and polymerase extension at 72°C for 75 s. qPCR were performed using the kit Absolute qPCR SYBR Green/ROX Master Mix (Thermo Scientific). Plasmid clones corresponding to the regions amplified by both primer sets were used as positive controls and for standard curves (10-fold dilution series) in the qPCR assays. Plasmid DNA was linearized with *Hind*III and quantified using the Picogreen dsDNA assay (Invitrogen, Eugene, OR). Extracted DNAs were normalized to 0.5 and 1 ng µL^{-1}. qPCR reactions were performed

in a total reaction volume of 10 µl using the SYBR Green/ROX qPCR Master Mix kit (Applied Biosystems) using 96-well plates. qPCR assays were performed in triplicates using a Chromo4 Real-Time PCR Detection System and the Opticon Monitor software package (Bio-Rad) with the following thermal cycling conditions: 95°C for 15 min, 45 cycles of denaturation at 95°C for 45 s, annealing temperature at 63°C for 45 s with fluorescence measurements after this step, and polymerase extension at 72°C for 15 s. Melting curves were generated after each assay to check the specificity of the amplification by heating from 65 to 95°C and taking fluorescence measurements every 0.5°C. Only single peaks were observed in the dissociation curves for both the standards and samples, indicating specific amplification with each set of primers. Negative controls consisted in three blanks, using the same PCR mix but without DNA template. Average amplification efficiencies were as follows: *P. rostrata* primer set = 100% and *P. infectans* primer set= 98%.

Genotyping and determination of genetic clusters

Genetic differentiation among host strains was analyzed with STRUCTURE version 2.2 (Pritchard *et al.* 2000), allowing classification into three genetic clusters.

For this analysis, only 9 microsatellite loci were retained in order to allow a maximum of 18% of missing data per locus. STRUCTURE uses a model-based Bayesian method to delineate clusters of individuals on the basis of their genotypes at multiple loci. Hardy-Weinberg and linkage disequilibrium are minimized within clusters. The estimated log probability of data $Pr(X \mid K)$ for each value of K is given, allowing the estimation of the more likely number of clusters. The analysis was run on the whole data set without any a priori population assignments and admixture was allowed. For each value of the number of cluster K (all values from 1 to 11), we ran 20 simulations using a burn-in of 100,000 iterations and a run length of 1,000,000 iterations. The best K was inferred both from the posterior probability of the data, L(K), and following the ΔK choice criterion of (Evanno *et al.* 2005). For the selected K, results from the 20 replicate runs were averaged using CLUMPP version 1.1.2 (Jakobsson & Rosenberg 2007) and each strains was assigned to a genetic cluster depending on their probability of assignment (> 55%).

Local adaptation

Local adaptation could be tested only for the parasite *P. infectans*, as *P. rostrata* is present only in the Penzé estuary. To make meaningful comparisons across estuaries, we set the time of maximum *A. minutum* density to day 0 in both estuaries (i.e., day 0 is 22 June 2011 in Penzé, and 27 May 2011 in Rance). Local adaptation was defined in two ways. When two populations are assayed, it has been suggested that local adaptation is present only under the strict criterion when the reaction norms across environments cross (Kawecki & Ebert 2004). Additionally we defined local adaptation as the difference between fitness in sympatry and fitness in allopatry. However, when only two demes are sampled, this contrast could represent any form of interaction between population and habitat, and not necessarily local adaptation. Moreover, with two demes it is not possible to test for the significance of this contrast while accounting for habitat and population main effects (Blanquart *et al.* 2013). To resolve the latter problem, we tested significance of the sympatric versus allopatric (SA) contrast using a permutation test. Specifically, we generated a null distribution of the SA contrast by considering the same transfers as for the true SA contrast, but where the sympatric versus allopatric status of transfers were randomized. This test can be thought of as a test of non-parallelism of reaction norms.

Patterns of local adaptation can be more apparent when the parasites are tested with hosts time-shifted in the past (Koskella 2014). We first tested local adaptation using all transfers where the parasite and host populations were sampled after day 0. Second we measured a time-shifted local adaptation where we tested the parasite populations sampled after day 0, and the host populations sampled at or before day 0.

Because parasites from Rance were generally less infectious than parasites from Penzé, the stricter criterion (reaction norm crossing) was not fulfilled (Sup. Fig. 1). Nevertheless, our analysis revealed a pattern of non-parallelism, whereby the fraction of host strains infected by the parasite in allopatry is 0.18 greater than in sympatry (p = 0.042, using a permutation test with 1000 permutations). In contrast, when considering time-shifted local adaptation, using the hosts sampled at or before day 0, the parasite local adaptation was positive at 0.13 (p = 0.068) (Sup. Table 5). These

contrasted patterns of non-parallelism cannot be interpreted as local adaptation, but mainly emerged as the parasite in Rance, in sympatry, had quite low infectivity on the contemporaneous host (host sampled after day 0), but slightly larger infectivity on the hosts of the past (hosts sampled before day 0), as the host in Rance evolved an increased resistance (see analysis of temporal trends, Fig. 4).

Correcting the significance levels in the generalized linear model using randomized pseudo-datasets

Sampling of parasite strains was relatively limited (at several dates only 1 strain was available), which hinders detection of temporal trends if the parasite population exhibits large variability. In particular the limited number of strains sampled at each time point is not necessarily representative of the true variability within the population, such that variation across time points attributed to temporal variations could in fact be due to sampling effects. We used simulation to determine the type 1 error rate of the model (rate of false positive) for the detection of temporal trends in infectivity or resistance. Specifically we used randomized pseudo-datasets with exactly the same structure as the true dataset but where the host and parasite strains were randomized. These randomized pseudo-datasets comply to the null hypothesis (no difference in infectivity between date host and date parasite), and if the linear model behaves ideally, the type I error rate on randomized pseudo-datasets should be equal to the significance level (here, 0.05). This randomization test revealed that the limited sampling of parasite strains leads to type I error at a rate that is sometimes higher than the expected 0.05. We therefore used the distribution of p-values on 500 pseudo-datasets to adjust the significance level for the temporal effects on the true dataset, ensuring a proper estimation of p-values.

Supplementary Table 1. Coordinates of fixed stations sampled in the Rance estuary.

Stations	Latitude	Longitude
1	48°30'00.15"	1°58'49.56"
2	48°30'11.00"	1°58'50.94"
3	48°30'30.48"	1°58'59.71"
4	48°31'24.69"	1°58'52.58"
5	48°32'07.51"	1°58'19.33"
6	48°32'45.32"	1°58'17.30"
7	48°32'03.58"	1°58'11.00"
8	48°30'52.14"	1°58'35.62"
9	48°29'51.67"	1°59'11.64"
10	48°29'49.29"	1°59'36.86"

Supplementary Table 2. List of host and parasite strains used in this study. RCC: Roscoff Culture Collection.

Species	Host genotype clusters	Use in this study	RCC number	**In** (Dia *et al.* 2014)	Synonymous	Ecosystems	Date of isolation
Alexandrium minutum	2	Cross-infections	RCC 3250	*	PARALEX 575	Penzé	01/06/2011
Alexandrium minutum	1	Cross-infections	RCC 3251	*	PARALEX 577	Penzé	01/06/2011
Alexandrium minutum	3	Cross-infections	RCC 3253	*	PARALEX 579	Penzé	01/06/2011
Alexandrium minutum	2	Cross-infections	RCC 3255	*	PARALEX 581	Penzé	01/06/2011
Alexandrium minutum	3	Cross-infections	RCC 3256	*	PARALEX 582	Penzé	01/06/2011
Alexandrium minutum	3	Cross-infections	RCC 3257	*	PARALEX 583	Penzé	01/06/2011
Alexandrium minutum	1	Cross-infections	RCC 3258	*	PARALEX 584	Penzé	01/06/2011
Alexandrium minutum	1	Cross-infections	RCC 3259	*	PARALEX 586	Penzé	01/06/2011
Alexandrium minutum	3	Cross-infections	RCC 3260	*	PARALEX 587	Penzé	01/06/2011
Alexandrium minutum	1	Cross-infections	RCC 3261	*	PARALEX 589	Penzé	01/06/2011
Alexandrium minutum	2	Cross-infections	RCC 3262	*	PARALEX 590	Penzé	01/06/2011
Alexandrium minutum	3	Cross-infections	RCC 3263	*	PARALEX 591	Penzé	01/06/2011
Alexandrium minutum	2	Cross-infections	RCC 3264	*	PARALEX 593	Penzé	01/06/2011
Alexandrium minutum	3	Cross-infections	RCC 3265	*	PARALEX 594	Penzé	01/06/2011
Alexandrium minutum	2	Cross-infections	RCC 3267	*	PARALEX 597	Penzé	01/06/2011
Alexandrium minutum	1	Cross-infections			PARALEX 619	Penzé	09/06/2011
Alexandrium minutum	3	Cross-infections			PARALEX 622	Penzé	09/06/2011
Alexandrium minutum	ND	Cross-infections			PARALEX 623	Penzé	09/06/2011
Alexandrium minutum	ND	Cross-infections			PARALEX 624	Penzé	09/06/2011
Alexandrium minutum	1	Cross-infections			PARALEX 626	Penzé	09/06/2011
Alexandrium minutum	ND	Cross-infections			PARALEX 627	Penzé	09/06/2011
Alexandrium minutum	1	Cross-infections			PARALEX 628	Penzé	09/06/2011
Alexandrium minutum	ND	Cross-infections			PARALEX 630	Penzé	09/06/2011
Alexandrium minutum	ND	Cross-infections			PARALEX 633	Penzé	09/06/2011
Alexandrium minutum	1	Cross-infections			PARALEX 635	Penzé	09/06/2011
Alexandrium minutum	3	Cross-infections	RCC 3326	*	PARALEX 753	Penzé	22/06/2011
Alexandrium minutum	3	Cross-infections	RCC 3327	*	PARALEX 754	Penzé	22/06/2011
Alexandrium minutum	1	Cross-infections	RCC 3331	*	PARALEX 761	Penzé	22/06/2011
Alexandrium minutum	1	Cross-infections	RCC 3333	*	PARALEX 764	Penzé	22/06/2011
Alexandrium minutum	ND	Cross-infections			PARALEX 765	Penzé	22/06/2011
Alexandrium minutum	2	Cross-infections	RCC 3334	*	PARALEX 766	Penzé	22/06/2011
Alexandrium minutum	2	Cross-infections	RCC 3335	*	PARALEX 767	Penzé	22/06/2011
Alexandrium minutum	2	Cross-infections	RCC 3336	*	PARALEX 770	Penzé	22/06/2011
Alexandrium minutum	ND	Cross-infections	RCC 3337	*	PARALEX 771	Penzé	22/06/2011
Alexandrium minutum	ND	Cross-infections			PARALEX 774	Penzé	22/06/2011
Alexandrium minutum	1	Cross-infections	RCC 3339	*	PARALEX 775	Penzé	22/06/2011
Alexandrium minutum	2	Cross-infections	RCC 3342	*	PARALEX 780	Penzé	22/06/2011
Alexandrium minutum	2	Cross-infections	RCC 3343	*	PARALEX 781	Penzé	22/06/2011
Alexandrium minutum	ND	Cross-infections	RCC 3348	*	PARALEX 788	Penzé	22/06/2011
Alexandrium minutum	1	Cross-infections	RCC 3349	*	PARALEX 789	Penzé	22/06/2011
Alexandrium minutum	3	Cross-infections			PARALEX 821	Penzé	30/06/2011
Alexandrium minutum	1	Cross-infections			PARALEX 822	Penzé	30/06/2011
Alexandrium minutum	3	Cross-infections			PARALEX 825	Penzé	30/06/2011

Alexandrium minutum	ND	Cross-infections			PARALEX 828	Penzé	30/06/2011
Alexandrium minutum	ND	Cross-infections			PARALEX 830	Penzé	30/06/2011
Alexandrium minutum	ND	Cross-infections			PARALEX 831	Penzé	30/06/2011
Alexandrium minutum	ND	Cross-infections			PARALEX 832	Penzé	30/06/2011
Alexandrium minutum	ND	Cross-infections			PARALEX 833	Penzé	30/06/2011
Alexandrium minutum	ND	Cross-infections			PARALEX 838	Penzé	30/06/2011
Alexandrium minutum	ND	Cross-infections			PARALEX 840	Penzé	30/06/2011
Alexandrium minutum	ND	Cross-infections			PARALEX 841	Penzé	30/06/2011
Alexandrium minutum	ND	Cross-infections			PARALEX 843	Penzé	30/06/2011
Alexandrium minutum	ND	Cross-infections			PARALEX 844	Penzé	30/06/2011
Alexandrium minutum	ND	Cross-infections			PARALEX 1091	Penzé	30/06/2011
Alexandrium minutum	2	Cross-infections	RCC 3352	*	PARALEX 640	Penzé	11/07/2011
Alexandrium minutum	2	Cross-infections	RCC 3353	*	PARALEX 641	Penzé	11/07/2011
Alexandrium minutum	3	Cross-infections	RCC 3354	*	PARALEX 643	Penzé	11/07/2011
Alexandrium minutum	3	Cross-infections	RCC 3356	*	PARALEX 645	Penzé	11/07/2011
Alexandrium minutum	2	Cross-infections	RCC 3357	*	PARALEX 646	Penzé	11/07/2011
Alexandrium minutum	2	Cross-infections	RCC 3358	*	PARALEX 647	Penzé	11/07/2011
Alexandrium minutum	3	Cross-infections	RCC 3359	*	PARALEX 648	Penzé	11/07/2011
Alexandrium minutum	3	Cross-infections	RCC 3360	*	PARALEX 655	Penzé	11/07/2011
Alexandrium minutum	1	Cross-infections	RCC 3361	*	PARALEX 658	Penzé	11/07/2011
Alexandrium minutum	2	Cross-infections	RCC 3362	*	PARALEX 660	Penzé	11/07/2011
Alexandrium minutum	3	Cross-infections	RCC 3363	*	PARALEX 661	Penzé	11/07/2011
Alexandrium minutum	ND	Cross-infections			PARALEX 664	Penzé	11/07/2011
Alexandrium minutum	1	Cross-infections	RCC 3364	*	PARALEX 665	Penzé	11/07/2011
Alexandrium minutum	ND	Cross-infections			PARALEX 676	Penzé	11/07/2011
Alexandrium minutum	ND	Cross-infections			PARALEX 678	Penzé	11/07/2011
Alexandrium minutum	3	Cross-infections	RCC 3365	*	PARALEX 679	Penzé	11/07/2011
Alexandrium minutum	1	Cross-infections	RCC 3366	*	PARALEX 682	Penzé	11/07/2011
Alexandrium minutum	1	Cross-infections	RCC 3367	*	PARALEX 683	Penzé	11/07/2011
Alexandrium minutum	1	Cross-infections	RCC 3186		PARALEX 919	Rance	09/05/2011
Alexandrium minutum	1	Cross-infections	RCC 3188		PARALEX 922	Rance	09/05/2011
Alexandrium minutum	ND	Cross-infections			PARALEX 924	Rance	09/05/2011
Alexandrium minutum	2	Cross-infections	RCC 3189	*	PARALEX 925	Rance	09/05/2011
Alexandrium minutum	3	Cross-infections	RCC 3190	*	PARALEX 926	Rance	09/05/2011
Alexandrium minutum	3	Cross-infections	RCC 3191	*	PARALEX 927	Rance	09/05/2011
Alexandrium minutum	3	Cross-infections	RCC 3192	*	PARALEX 930	Rance	09/05/2011
Alexandrium minutum	2	Cross-infections	RCC 3193	*	PARALEX 931	Rance	09/05/2011
Alexandrium minutum	1	Cross-infections	RCC 3194	*	PARALEX 932	Rance	09/05/2011
Alexandrium minutum	1	Cross-infections	RCC 3195	*	PARALEX 934	Rance	09/05/2011
Alexandrium minutum	ND	Cross-infections	RCC 3196	*	PARALEX 937	Rance	09/05/2011
Alexandrium minutum	2	Cross-infections	RCC 3197	*	PARALEX 938	Rance	09/05/2011
Alexandrium minutum	2	Cross-infections	RCC 3224	*	PARALEX 1019	Rance	27/05/2011
Alexandrium minutum	2	Cross-infections	RCC 3227	*	PARALEX 1022	Rance	27/05/2011
Alexandrium minutum	1	Cross-infections	RCC 3228	*	PARALEX 1024	Rance	27/05/2011
Alexandrium minutum	ND	Cross-infections			PARALEX 1025	Rance	27/05/2011
Alexandrium minutum	2	Cross-infections	RCC 3230	*	PARALEX 1028	Rance	27/05/2011
Alexandrium minutum	1	Cross-infections	RCC 3233	*	PARALEX 1035	Rance	27/05/2011
Alexandrium minutum	ND	Cross-infections			PARALEX 1037	Rance	27/05/2011

Alexandrium minutum	ND	Cross-infections			PARALEX 1038	Rance	27/05/2011
Alexandrium minutum	3	Cross-infections	RCC 3235	*	PARALEX 1039	Rance	27/05/2011
Alexandrium minutum	2	Cross-infections	RCC 3236	*	PARALEX 1040	Rance	27/05/2011
Alexandrium minutum	ND	Cross-infections			PARALEX 1041	Rance	27/05/2011
Alexandrium minutum	2	Cross-infections	RCC 3237	*	PARALEX 1042	Rance	27/05/2011
Alexandrium minutum	2	Cross-infections	RCC 3239	*	PARALEX 1044	Rance	27/05/2011
Alexandrium minutum	ND	Cross-infections			PARALEX 1045	Rance	27/05/2011
Alexandrium minutum	ND	Cross-infections			PARALEX 1046	Rance	27/05/2011
Alexandrium minutum	ND	Cross-infections	RCC 3278	*	PARALEX 873	Rance	05/06/2011
Alexandrium minutum	2	Cross-infections	RCC 3279	*	PARALEX 874	Rance	05/06/2011
Alexandrium minutum	1	Cross-infections	RCC 3280	*	PARALEX 875	Rance	05/06/2011
Alexandrium minutum	3	Cross-infections	RCC 3281	*	PARALEX 876	Rance	05/06/2011
Alexandrium minutum	1	Cross-infections	RCC 3282	*	PARALEX 877	Rance	05/06/2011
Alexandrium minutum	2	Cross-infections	RCC 3283	*	PARALEX 878	Rance	05/06/2011
Alexandrium minutum	ND	Cross-infections			PARALEX 879	Rance	05/06/2011
Alexandrium minutum	1	Cross-infections	RCC 3284	*	PARALEX 880	Rance	05/06/2011
Alexandrium minutum	1	Cross-infections	RCC 3285	*	PARALEX 881	Rance	05/06/2011
Alexandrium minutum	ND	Cross-infections	RCC 3286	*	PARALEX 882	Rance	05/06/2011
Alexandrium minutum	3	Cross-infections	RCC 3287	*	PARALEX 883	Rance	05/06/2011
Alexandrium minutum	1	Cross-infections	RCC 3288	*	PARALEX 884	Rance	05/06/2011
Alexandrium minutum	ND	Cross-infections	RCC 3289	*	PARALEX 885	Rance	05/06/2011
Alexandrium minutum	3	Cross-infections	RCC 3290	*	PARALEX 886	Rance	05/06/2011
Alexandrium minutum	3	Cross-infections	RCC 3291	*	PARALEX 887	Rance	05/06/2011
Alexandrium minutum	ND	Cross-infections	RCC 3187	*	PARALEX 921	Rance	05/06/2011
Alexandrium minutum		Isolation of parasites	RCC 3018		AM89BM2	Morlaix	1989
Alexandrium minutum		Isolation of parasites	RCC 3019		AM99PZ2	Penzé	1999
Alexandrium minutum		Isolation of parasites	RCC 3020		PARALEX 178	Penzé	30/06/2007
Alexandrium minutum		Isolation of parasites	RCC 3022	*	PARALEX 292	Rance	02/06/2010
Alexandrium minutum		Isolation of parasites	RCC 2651		PARALEX 263	Rance	17/05/2010
Alexandrium minutum		Isolation of parasites	RCC 3023		PARALEX 524	Penzé	01/07/2010
Alexandrium minutum		Isolation of parasites	RCC 3024		PARALEX 381	Penzé	08/06/2010
Alexandrium minutum		Isolation of parasites	RCC 3026		PARALEX 157	Penzé	30/06/2007
Alexandrium minutum		Isolation of parasites	RCC 3027		PARALEX 465	Rance	22/06/2010
Alexandrium minutum		Isolation of parasites	RCC 3028		PARALEX 458	Penzé	20/06/2010
Alexandrium minutum		Isolation of parasites	RCC 3030		CBA38	Adriatic Sea	XX/05/2008
Alexandrium minutum		Isolation of parasites	RCC 3031		CBA42	Adriatic Sea	XXX
Alexandrium minutum		Isolation of parasites	RCC 3032		SZN30	Gulf of Naples	18/05/2000
Parvilucifera rostrata		Cross-infections	RCC 2861		PARALEX P163	Penzé	19/06/2011
Parvilucifera rostrata		Cross-infections	RCC 2862		PARALEX P164	Penzé	21/06/2011
Parvilucifera rostrata		Cross-infections	RCC 2829		PARALEX P131	Penzé	21/06/2011
Parvilucifera rostrata		Cross-infections	RCC 2916		PARALEX P218	Penzé	19/06/2011
Parvilucifera rostrata		Cross-infections	RCC 2893		PARALEX P195	Penzé	19/06/2011
Parvilucifera rostrata		Cross-infections	RCC 2876		PARALEX P178	Penzé	21/06/2011
Parvilucifera rostrata		Cross-infections	RCC 2885		PARALEX P187	Penzé	21/06/2011
Parvilucifera rostrata		Cross-infections	RCC 2917		PARALEX P219	Penzé	21/06/2011
Parvilucifera rostrata		Cross-infections	RCC 2827		PARALEX P129	Penzé	19/06/2011
Parvilucifera rostrata		Cross-infections	RCC 2823		PARALEX P124	Penzé	01/07/2011
Parvilucifera rostrata		Cross-infections	RCC 2839		PARALEX P141	Penzé	30/06/2011

Parvilucifera rostrata	Cross-infections	RCC 2842	PARALEX P144	Penzé	01/07/2011
Parvilucifera rostrata	Cross-infections	RCC 2849	PARALEX P151	Penzé	30/06/2011
Parvilucifera rostrata	Cross-infections	RCC 2850	PARALEX P152	Penzé	30/06/2011
Parvilucifera rostrata	Cross-infections	RCC 2888	PARALEX P190	Penzé	01/07/2011
Parvilucifera rostrata	Cross-infections	RCC 2891	PARALEX P193	Penzé	30/06/2011
Parvilucifera rostrata	Cross-infections	RCC 2907	PARALEX P209	Penzé	30/06/2011
Parvilucifera rostrata	Cross-infections	RCC 2913	PARALEX P215	Penzé	30/06/2011
Parvilucifera rostrata	Cross-infections	RCC 2926	PARALEX P228	Penzé	29/06/2011
Parvilucifera rostrata	Cross-infections	RCC 2927	PARALEX P229	Penzé	29/06/2011
Parvilucifera rostrata	Cross-infections	RCC 2928	PARALEX P230	Penzé	30/06/2011
Parvilucifera rostrata	Cross-infections	RCC 2929	PARALEX P231	Penzé	08/07/2011
Parvilucifera infectans	Cross-infections	RCC 2824	PARALEX P126	Penzé	02/07/2011
Parvilucifera infectans	Cross-infections	RCC 2870	PARALEX P172	Penzé	27/06/2011
Parvilucifera infectans	Cross-infections	RCC 2902	PARALEX P204	Penzé	26/06/2011
Parvilucifera infectans	Cross-infections	RCC 2922	PARALEX P224	Penzé	23/06/2011
Parvilucifera infectans	Cross-infections	RCC 2903	PARALEX P205	Penzé	26/06/2011
Parvilucifera infectans	Cross-infections	RCC 2837	PARALEX P139	Penzé	27/06/2011
Parvilucifera infectans	Cross-infections	RCC 2901	PARALEX P203	Penzé	25/06/2011
Parvilucifera infectans	Cross-infections	RCC 2867	PARALEX P169	Penzé	26/06/2011
Parvilucifera infectans	Cross-infections	RCC 2857	PARALEX P159	Rance	08/06/2011
Parvilucifera infectans	Cross-infections	RCC 2853	PARALEX P155	Rance	04/06/2011
Parvilucifera infectans	Cross-infections	RCC 2826	PARALEX P128	Rance	05/06/2011
Parvilucifera infectans	Cross-infections	RCC 2879	PARALEX P181	Rance	08/06/2011
Parvilucifera infectans	Cross-infections	RCC 2912	PARALEX P214	Rance	15/06/2011
Parvilucifera infectans	Cross-infections	RCC 2819	PARALEX P120	Rance	08/06/2011
Parvilucifera infectans	Cross-infections	RCC 2852	PARALEX P154	Rance	08/06/2011

Supplementary Table 3. Characteristics of host-parasite networks. H = number of host strains. P = number of parasite strains. I = number of interactions (total of positive infection). NA= number of none acquired data. M = size (M=H*P-NA). C = infectivity (or connectance, C=I/M). LP = average number of interactions across parasite strains (LP=I/P). PZ, Penzé; RC, Rance.

	H	P	I	NA	M	C	LP
***Parvilucifera rostrata* PZ**							
**Sympatry (host from PZ)**							
01/06/2011	15	22	244	1	329	0.74	11.09
09/06/2011	10	14	125	0	140	0.89	8.93
22/06/2011	15	22	274	0	330	0.83	12.45
30/06/2011	14	14	171	0	196	0.87	12.21
11/07/2011	18	22	319	0	396	0.81	14.50
Average Sympatry						0.83	11.84
Median Sympatry						0.83	12.21
**Allopatry (host from RC)**							
09/05/2011	12	14	146	0	168	0.87	10.43
27/05/2011	15	14	180	0	210	0.86	12.86
05/06/2011	16	14	189	0	224	0.84	13.50
Average Allopatry						0.86	12.26
Median Allopatry						0.86	12.86
Total Average						***0.84***	***12.00***
Total Median						***0.85***	***12.33***
***Parvilucifera infectans* PZ**							
**Sympatry (host from PZ)**							
01/06/2011	15	8	90	3	117	0.77	11.25
09/06/2011	10	8	61	0	80	0.76	7.63
22/06/2011	15	8	103	0	120	0.86	12.88
30/06/2011	14	8	72	0	112	0.64	9.00
11/07/2011	18	8	103	0	144	0.72	12.88
Average Sympatry						0.75	10.73
Median Sympatry						0.76	11.25
**Allopatry (host from RC)**							
09/05/2011	12	8	63	0	96	0.66	7.88
27/05/2011	15	8	101	1	119	0.85	12.63
05/06/2011	16	8	84	0	128	0.66	10.50
Average Allopatry						0.72	10.33
Median Allopatry						0.66	10.50
Total Average						***0.74***	***10.58***
Total Median						***0.74***	***10.88***
***Parvilucifera infectans* RC**							
**Sympatry (host from RC)**							
09/05/2011	12	7	32	0	84	0.38	4.57
27/05/2011	15	7	44	2	103	0.43	6.29
05/06/2011	16	7	24	1	111	0.22	3.43
Average Sympatry						0.34	4.76
Median Sympatry						0.38	4.57

**Allopatry (host from PZ)**							
01/06/2011	15	7	34	0	105	0.32	4.86
09/06/2011	10	7	33	0	70	0.47	4.71
22/06/2011	15	7	30	0	105	0.29	4.29
30/06/2011	14	7	35	0	98	0.36	5.00
11/07/2011	18	7	47	0	126	0.37	6.71
Average Allopatry						0.36	5.11
Median Allopatry						0.36	4.86
Total Average						***0.35***	***4.98***
Total Median						***0.37***	***4.79***
**Total number of cross-infections**					**3711**		

Supplementary Table 4. Infectivity matrices are significantly nested. For each matrix, we calculate temperature, an indicator of nestedness, using the program BINMATNEST, implemented in R (package bipartite, (Rodríguez-Gironés & Santamaría 2006)). The p-value is the probability that the same temperature arises under the null model where average infectivity is the same, but where host strain x parasite strain combinations leading to infection are randomly chosen in the matrix. This probability was computed using 10^3 randomized matrices. PZ, Penzé; RC, Rance.

Parasite, hosts	Number of parasite strains	Number of host strains	Average infectivity	Temperature	p-value
P. infectans PZ, host PZ	8	72	0.75	8.9	0
P. infectans RC, host RC	7	33	0.34	26.9	0.002
P. rostrata PZ, host PZ	22	72	0.81	16	0

Supplementary Table 5. Local adaptation tested using reciprocal experimental design on *P. infectans*. LA = mean infectivity obtained with cross-infections in sympatry (PZ*PZ + RC*RC) – mean infectivity obtained with cross-infections in allopatry (PZ*RC + RC*PZ). PZ=Penzé and RC=Rance. For the first row, all host and parasite strains sampled after time 0 were used. For the second row, we used the same parasite strains as for the first row, but used time-shifted host strains sampled before time 0 (time 0 is defined for each estuary as the date of maximum density of the host *A. minutum*).

	LA	Permutation test
host and parasite > 0	-0.175	0.042
host <= 0, parasite > 0	0.128	0.068

Supplementary Tables 6. Summary tables of the generalized linear mixed model explaining infection outcome (binary variable, 1 or 0) as a function of an intercept, host date, parasite date, host cluster (1, 2, 3 or unknown), the time shift effect ("far past", "near past", "present", "near future"), a random effect for host strain representing host genetic variability for resistance, and a random effect for parasite strain representing parasite genetic variability for infectivity. All effects are presented on the logit scale. 95% confidence intervals were computed using a parametric bootstrap with resampling of residual errors. For host and parasite date, effects were computed relative to the earliest date; for host cluster, relative to cluster 1; for time-shift, relative to "far past". p-values for the fixed effects (date host, date parasite, host cluster and time shift) as well as the random effects (host strain, parasite strain) were computed using a type II analysis of deviance based on chisquare tests. p-values for "date host", "date parasite", and "time shift" were corrected using the distribution of p-values obtained in datasets where strains were randomized with respect to date. This ensures the reported p-values are uniformly distributed on [0, 1] under the null hypothesis where there is no temporal variation in infectivity and resistance. Furthermore, for date host and date parasite we also compute p-values based on an analysis of variance on the data averaged over all parasite strains (for hosts) or all host strains (for parasites) (the second p-value reported).

**P. infectans from Rance with Rance host**			
**Effect**	**Estimate and 95% CI**	**p-value**	
Intercept	-2.29 [-3.44; -1.5]	-	-
Host Date 0	0.0981 [-1.12; 1.15]	0.082	0.092
Host Date 9	-2.12 [-3.76; -0.889]		
Parasite Date 9	1.1 [-0.017; 2.26]	0.41	0.68
Parasite Date 12	1.64 [0.944; 2.78]		
Parasite Date 19	2.5 [1.33; 4.04]		
Host Cluster 2	-0.935 [-1.84; -0.0148]	0.33	
Host Cluster 3	0.445 [-0.412; 1.23]		
Host Cluster NA	0.283 [-0.555; 1.04]		
Time Shift near past	0.408 [-0.676; 1.57]	0.62	
Time Shift present	1.09 [-0.25; 2.92]		
Host Strain Variance	2.01 [0.351; 2.39]	9.10e-08	
Parasite Strain Variance	0.284 [1.81e-06; 0.489]	0.021	

P. infectans from Penzé with Penzé host			
**Effect**	**Estimate and 95% CI**	**p-value**	
Intercept	0.897 [-0.121; 1.92]	-	-
Host Date -13	-0.104 [-0.962; 0.775]	0.024	0.00088
Host Date 0	0.12 [-0.956; 1.16]		
Host Date 8	-1.75 [-2.77; -0.454]		
Host Date 19	-0.134 [-1.28; 1.76]		
Parasite Date 3	21 [19.2; 23.2]	0.34	0.63
Parasite Date 4	1.04 [0.345; 1.93]		
Parasite Date 5	-0.646 [-1.32; 0.221]		
Parasite Date 10	-0.584 [-1.26; 0.517]		
Host Cluster 2	0.0955 [-0.585; 0.849]	0.97	
Host Cluster 3	0.17 [-0.489; 0.912]		
Host Cluster NA	0.119 [-0.528; 0.881]		
Time Shift near past	0.183 [-0.994; 1.31]	0.06	
Time Shift present	0.989 [-0.577; 2.31]		
Time Shift near future	-0.4 [-2.42; 0.773]		
Host Strain Variance	6.88e-07 [1.41e-08; 0.213]	1	
Parasite Strain Variance	0.759 [0.359; 1.3]	6.70e-12	

P. rostrata from Penzé with Penzé host			
**Effect**	**Estimate and 95% CI**	**p-value**	
Intercept	-1.45 [-2.25; -0.541]	-	-
Host Date -13	0.478 [-0.17; 1.29]	0.12	0.0058
Host Date 0	1.11 [0.0971; 2.11]		
Host Date 8	0.552 [-0.459; 1.68]		
Host Date 19	0.0754 [-0.929; 1.12]		
Parasite Date -1	0.548 [-0.153; 1.03]	0.062	0.28
Parasite Date 7	0.623 [-0.256; 1.37]		
Parasite Date 8	1.59 [0.903; 2.13]		
Parasite Date 9	6.48 [5.55; 8.65]		
Parasite Date 16	0.381 [-0.648; 1.38]		
Host Cluster 2	0.0863 [-0.343; 0.62]	0.73	
Host Cluster 3	0.382 [-0.0924; 0.811]		
Host Cluster NA	0.281 [-0.211; 0.824]		
Time Shift near past	1.97 [1.05; 2.99]	0.096	
Time Shift present	1.54 [0.325; 2.6]		
Time Shift near future	2.33 [0.894; 3.75]		
Host Strain Variance	0.63 [0.0375; 0.466*]	1.70e-06	
Parasite Strain Variance	2.29 [1.19; 2.58]	8.70e-40	

* The upper confidence limit for the genetic variance was below the maximum likelihood estimate of 0.63. This indicates the bootstrap procedure is not adequate to estimate confidence intervals for the two random effects variances ("Host Strain" and "Parasite Strain"). This is perhaps because the bootstrap uses resampling of the residual errors, which may tend to decrease the variance and thus underestimate the variances of random effects.

References

1.Blanquart, F., Kaltz, O., Nuismer, S.L. & Gandon, S. (2013). A practical guide to measuring local adaptation. *Ecol. Lett.*, 16, 1195–1205.

2.Coats, D.W. & Bockstahler, K.R. (1994). Occurrence of the parasitic dinoflagellate *Amoebophrya ceratii* in Chesapeake Bay populations of *Gymnodinium sanguineum*. *J. Eukaryot. Microbiol.*, 41, 586–593.

3.Dia, A., Guillou, L., Mauger, S., Bigeard, E., Marie, D., Valero, M., *et al.* (2014). Spatiotemporal changes in the genetic diversity of harmful algal blooms caused by the toxic dinoflagellate *Alexandrium minutum*. *Mol. Ecol.*, 23, 549–560.

4.Evanno, G., Regnaut, S. & Goudet, J. (2005). Detecting the number of clusters of individuals using the software STRUCTURE: a simulation study. *Mol. Ecol.*, 14, 2611–2620.

5.Jakobsson, M. & Rosenberg, N.A. (2007). CLUMPP: A cluster matching and permutation program for dealing with label switching and multimodality in analysis of population structure. *Bioinformatics*, 23, 1801–1806.

6.Kawecki, T.J. & Ebert, D. (2004). Conceptual issues in local adaptation. *Ecol. Lett.*, 7, 1225–1241.

7.Koskella, B. (2014). Bacteria-phage interactions across time and space : merging local adaptation and time-shift experiments to understand phage evolution. *Am. Nat.*, 184, Suppl1:S9–21.

8.Lebret, K., Kritzberg, E.S., Figueroa, R. & Rengefors, K. (2012). Genetic diversity within and genetic differentiation between blooms of a microalgal species. *Environ. Microbiol.*, 14, 2395–2404.

9.Maguer, J.-F., Wafar, M., Madec, C., Morin, P. & Erard-Le Denn, E. (2004). Nitrogen and phosphorus requirements of an *Alexandrium minutum* bloom in the Penzé estuary, France. *Limnol. Oceanogr.*, 49, 1108–1114.

10.Moon-van der Staay, S.Y., De Wachter, R. & Vaulot, D. (2001). Oceanic 18S rDNA sequences from picoplankton reveal unsuspected eukaryotic diversity. *Nature*, 409, 607–610.

11.Pritchard, J.K., Stephens, M. & Donnelly, P. (2000). Inference of population structure using multilocus genotype data. *Genetics*, 155, 945–959.

12.Rodríguez-Gironés, M. a. & Santamaría, L. (2006). A new algorithm to calculate the nestedness temperature of presence-absence matrices. *J. Biogeogr.*, 33, 924–935.

13.Scholin, C.A. & Anderson, D.M. (1994). Identification of group-specific and strain-specific genetic- markers for globally distributed *Alexandrium* (Dinophyceae) .1. Rflp analysis of SSU ribosomal-rRNA Genes. *J. Phycol.*, 30, 744–754.

Supplementary Figure 1. Local adaptation pattern for the parasite *P. infectans*, using all host and parasite populations sampled after day 0 (top row), or using the same set of parasites with time-shifted hosts sampled at or before day 0 (bottom row).

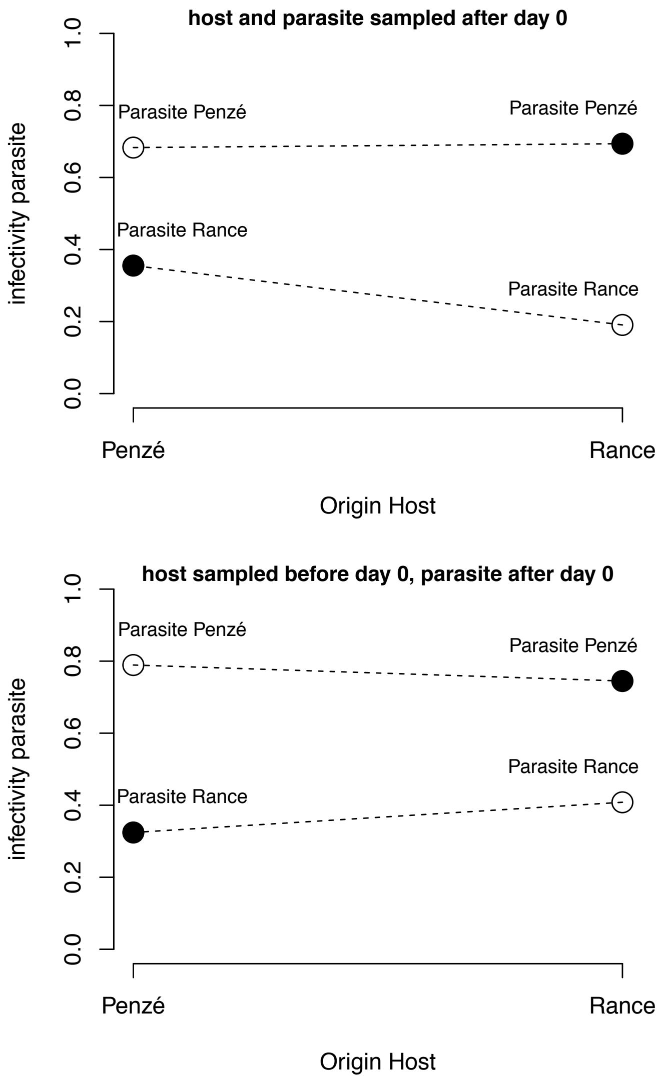

Supplement: Supplementary text and tables [file rspb20161870supp1.pdf]
